# Supplementary material for: The Role of Extracellular Polymeric Substances in the Toxicity Response of Anaerobic Granule Sludge to Different Metal Oxide Nanoparticles
Source: Int J Environ Res Public Health. 2022 Apr 28;19(9):5371. doi: 10.3390/ijerph19095371 (PMC9100327; doi:10.3390/ijerph19095371)
Supplement: Supplementary file 1 [file ijerph-19-05371-s001.zip › ijerph-1648002-supplementary.pdf]

Supplementary Information

S1. Materials and methods

S1.1 Synthetic Wastewater

To benefit the growth of the microbes in the AGS, glucose was selected as carbon source because it is easy to degrade. The synthetic medium composition contained glucose (7000 mg/L), nutrients (500 mg/L NH4Cl, 26 mg/L $K_2HPO_4 \bullet 3H_2O$, 0.25 g/L yeast extract, and 0.25 g/L tryptone), and trace elements based on the Vanderbilt mineral medium[1] except sulfate to avoid precipitation of Cu or Zn sulfides. The carbonate buffer (5.6 g/L $NaHCO_3$, 0.05 g/L $CaCl_2 \cdot 2H_2O$, and 0.2 g/L $MgCl_2 \cdot 6H_2O$) was also adopted to maintain the pH of the synthetic wastewater in a stable and controlled culture

S1.2 Pre-incubation of Methanogenic activity bioassays

The 1.08× concentrated medium (37 mL) which was the same as in the parent anaerobic reactor without glucose were added to each bottle under gas mixture N2/CO2 (80:20, v/v) atmosphere. After sieved though 70-mesh sieve to remove fine particles, the AGS withdrawn from the parent reactor t inoculated into the serum bottles to a final concentration of 1000 mgVSS/L. Either sodium acetate (1000 mgCOD/L) or H2 gas were used as substrate. H2 was supplied at an initial concentration of 0.5 atm of H2/CO2 (80:20, v/v) overpressure. Subsequently, all the assays were pre-incubated overnight avoid a lag phase caused by the atmospheric oxygen exposure.

S1.3 EPS extraction

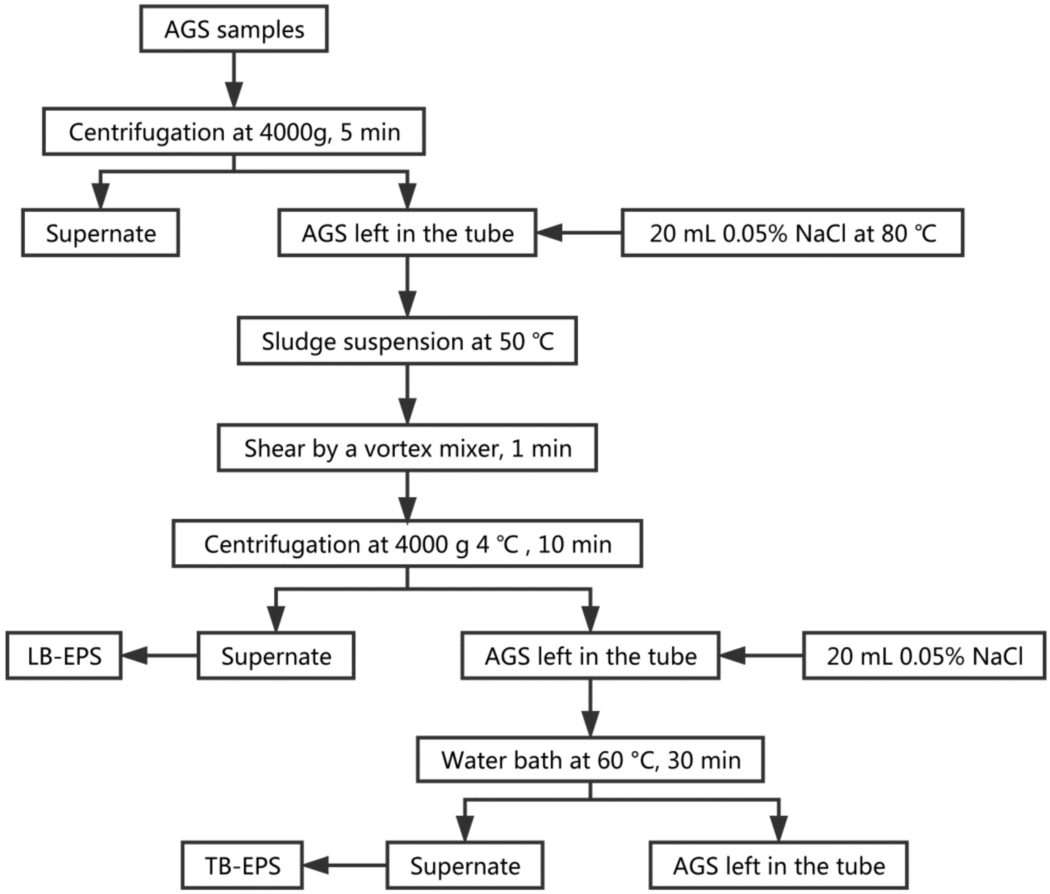

Figure S1. Methods for the extraction of AGS EPS.

S1.4 Fluorescence in situ hybridization (FISH)

The FISH analysis were performed following a modified protocol of Gomec, et al. [2], and carried out in three steps: fixation, hybridization and identification, as shown in **Figure S2** .

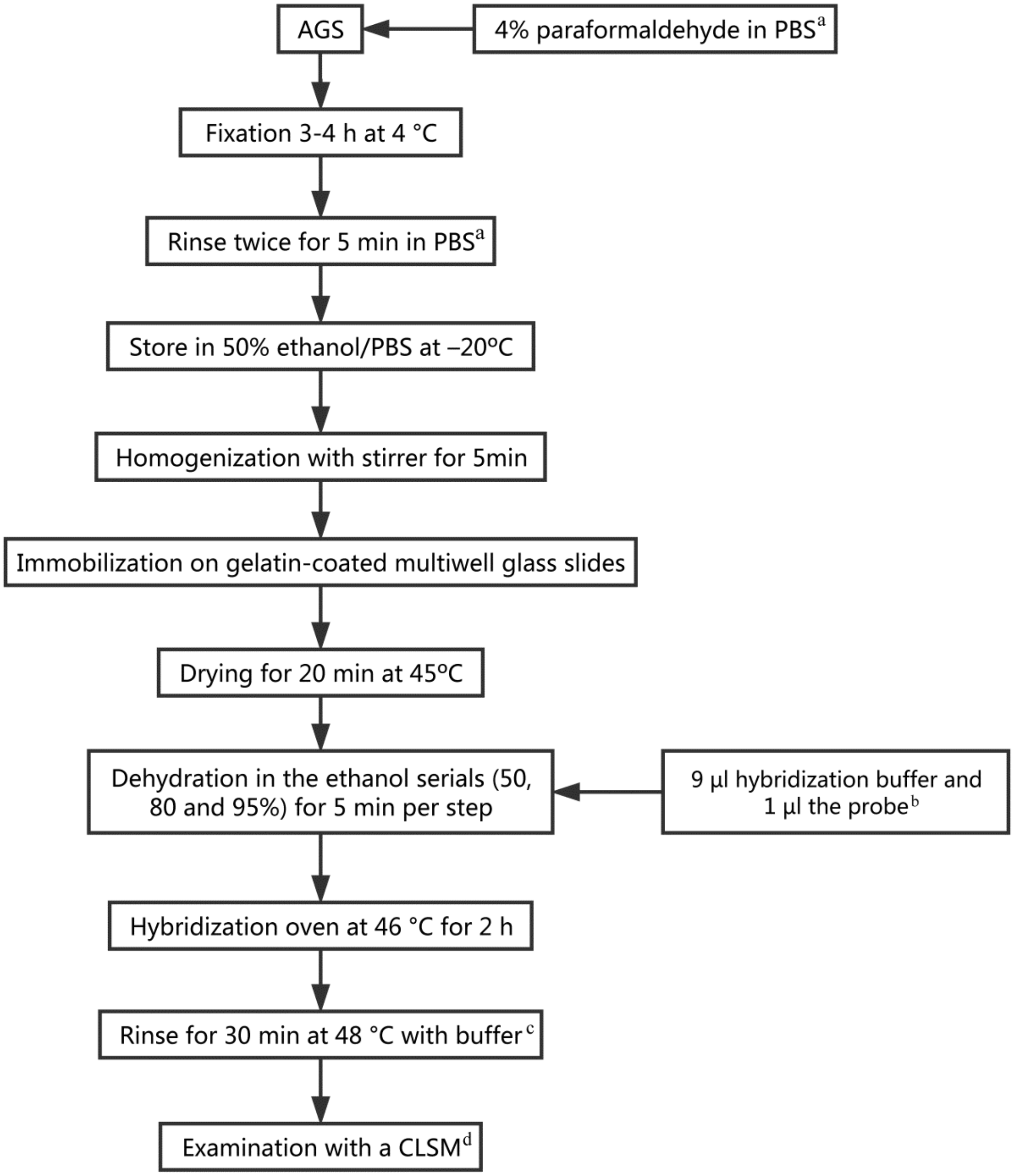

Figure S2. The protocol of FISH analysis. a The PBS components (pH 7.2): 0.13 M NaCl, 7mM Na_2HPO_4, 3 mM $Na_2H_2PO_4$. b The hybridization buffer components: 0.9 M NaCl, 20 mM Tris-HCl (pH 7.2), 0.01% SDS + 35% (ARC915) or 5% (EUB338) formamid; 5 ng/mL of the final probe concentration. c The buffer containing the same components as the hybridization buffer except for the probes. d Confocal laser scanning microscope (CLSM, FV10i, Olympus, Japan).

S1.4 Reactive oxygen species (ROS) production and lactate dehydrogenase (LDH) release

The production of intracellular ROS in AGS was by the DCF assay according to the reference [3]. Briefly, the AGS samples were first rinsed with 0.85% (w/w) NaCl solution three times. The pellets were then re-suspended in NaCl solution and incubated with 20 μM H2DCF-DA (Molecular Probes, Invitrogen, USA) at 35 ± 1 °C in darkness for 30 min. The harvested AGS by centrifugation was re-suspended in NaCl solution, and then plated in a 96-well plate (Molecular Devices, USA). Finally, the generated fluorescein DCF was measured by microplate reader (Synergy H1, Biotek, Vermont, USA) at excitation/emission wavelengths of 485/520 nm.

LDH was determined by using an LDH Assay Kit (Dojindo, Kumamoto, Japan) in accordance with the manufacturer's instructions: after the batch experiments, the supernatant was separated from the AGS by centrifugation at 12,000 g for 5 min and then transferred to mix with the working solution provided by the kit. After incubation at 37 ℃ for 30 min under dark condition, stop solution was added and the absorbance of mixture was measured at wavelength of 490 nm using a microplate reader (Synergy H1, Biotek, Vermont, USA).

Each of the above experiment was carried out in triplicate. ROS and LDH values of the treated groups were expressed as the percentage of control groups that were assumed to be 100%.

S1.5 NPs Fluorescent Labeling

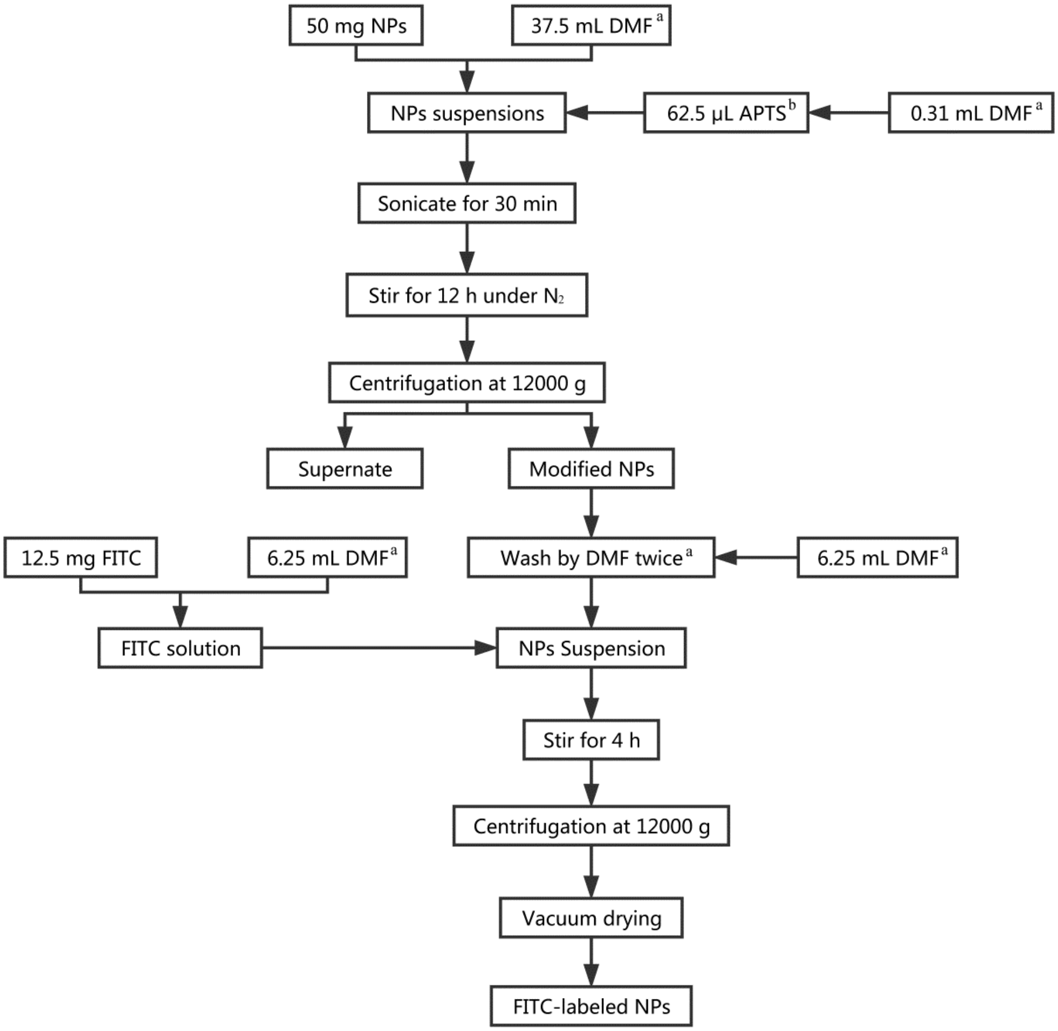

Figure S3. Procedures of NPs fluorescence labeling. ([a] DMF, anhydrous dimethylformamide. [b] APTS, aminopropyl triethoxy silane).

S1.6 Pretreatment of the AGS for SEM observation

The AGS samples were fixed overnight in 2.5% vol/vol glutaraldehyde (pH 7.2–7.4) at 4 °C, and then washed 3 times with 0.1M phosphate buffer solution for 15 min each. Subsequently, the specimens were dehydrated in a series of ethanol serials (25%–100%). After replacement by a mixture of isoamyl acetate and ethanol (volume ratio 1:1) and then pure isoamyl acetate for 15 min each, the pellets were dried with a critical-point drier and coated with platinum for SEM observations.

S2. Supplemental figures

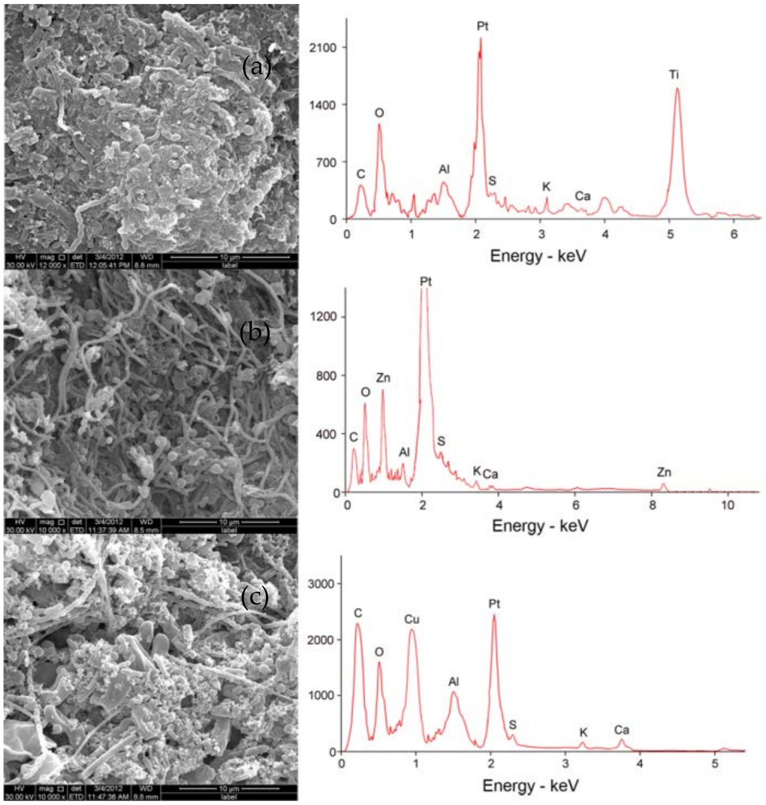

Figure S4. Typical SEM images and EDX analysis of AGS after TiO_2 NPs (a), ZnO NPs (b) and CuO NPs (c) exposure (200mg/gVSS).

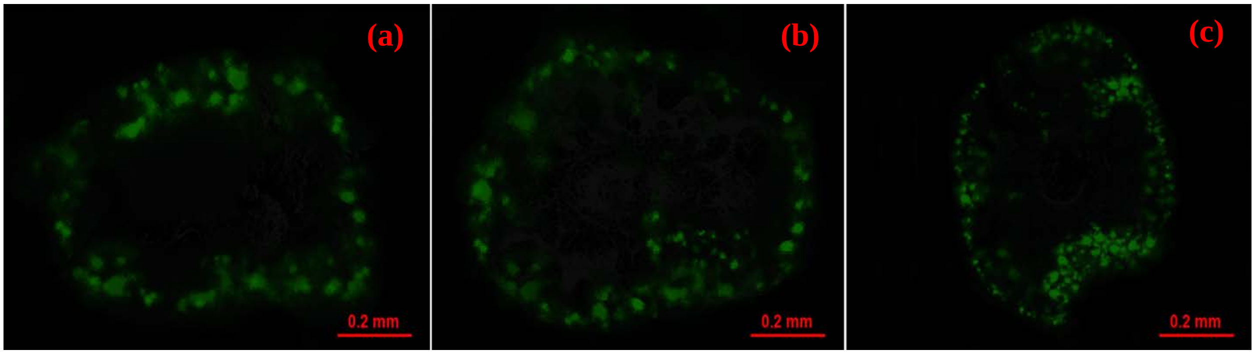

Figure S5. Distributions of FITC-labeled TiO_2 (a), ZnO (b) and CuO (c) NPs on AGS cross-section.

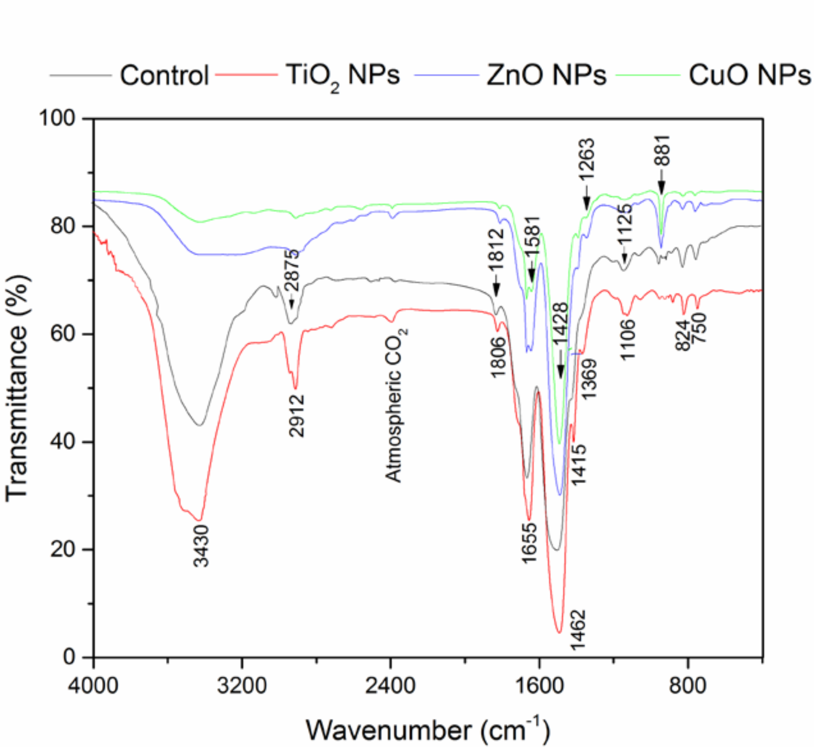

Figure S6. FTIR spectra of EPS in the absence and presence of 200 mg/gVSS of TiO_2, ZnO and CuO NPs. The results of FTIR analysis was shown in **Table S3**.

S3. Supplemental tables

Table S1. Effect of dispersant addition (10mg/L polyacrylamide) on the EPS and methane production under different NPs (200mg/L) exposure.

**NPs types**	**EPS production**						**Methane production**					
	No polyacrylamide (mg/gVSS)	Polyacrylamide (mg/gVSS)	T Test	DF [1]	Prob > |t| (p-value)	Significantly different [2]	No polyacrylamide (mL)	Polyacrylamide (mL)	T Test	DF [1]	Prob > |t| (p-value)	Significantly different [2]
Control	103.7 ± 4.5	104.2 ± 2.3	0.03	1	0.4	NO	24.4 ± 0.8	24.8 ± 0.9	0.4	1	0.4	NO
TiO_2	152.6 ± 6.6	149.2 ± 5.1	−0.2	1	0.2	NO	26.3 ± 0.6	25.9 ± 0.1	0.3	1	0.7	NO
ZnO	85.4 ± 3.8	92.5 ± 2.1	−1.1	1	0.2	NO	14.4 ± 0.3	15.2 ± 0.4	−1.6	1	0.5	NO
CuO	45.1 ± 1.4	48.9 ± 1.8	−0.9	1	0.8	NO	4.4 ± 0.1	4.9 ± 0.2	−0.5	1	0.5	NO

1 DF: Degrees of freedom.
2 Significance level = 0.05.

Table S2. Correlation analysis results of microbial abundance in AGS and individual compositions of EPS.

NPs type	Microbial	Correlation analysis results	EPS composition[a]		
			Protein	Polysaccharides	DNA
TiO$_2$	Bacteria	Spearman's correlation coefficient	−0.839	0.69	0.033
		Significance level	0.044	0.063	0.934
		N	9	9	9
	Archaea	Spearman's correlation coefficient	0.646	0.482	0.022
		Significance level	0.060	0.189	0.956
		N	9	9	9
ZnO	Bacteria	Spearman's correlation coefficient	−0.412	0.504	0.890
		Significance level	0.271	0.166	0.001
		N	9	9	9
	Archaea	Spearman's correlation coefficient	0.848	−0.470	−0.517
		Significance level	0.047	0.202	0.154
		N	9	9	9
CuO	Bacteria	Spearman's correlation coefficient	−0.761	−0.291	0.834
		Significance level	0.026	0.448	0.005
		N	9	9	9
	Archaea	Spearman's correlation coefficient	0.932	0.991	−0.724
		Significance level	2.56×10^{-4}	1.96×10^{-7}	0.027
		N	9	9	9

[a] All the statistically significant correlations have been highlighted in red.

Table S3. Main functional groups of EPS observed in the FT-IR spectra.

Wave number (cm^{-1})	Vibration type	Functional type
3430	–OH and –NH_2 stretching	Polysaccharides and proteins
2875–2912	CH_2 stretching	Aliphatic hydrocarbons
1806–1812	C = O stretching	VFAs

1655	C = O and C-N stretching (amide I)	Proteins (peptidic bond)
1581	C–N Stretching and N–H deformation (amide II)	Proteins (peptidic bond)
1415–1462	C-H bending and C-N stretching (amide III)	Proteins (peptidic bond)
1369	C = O stretching	Carboxylic groups
1263	C = O deformation	
1125–1136	C-O-C stretching	Polysaccharides
＜1000	Fingerprint region	Phosphate group

References

1. RE, S., Anaerobic biotechnology for industrial wastewater treatment. *Environ Sci Technol* **1983,** *17*, 416A–427A.
2. Gomec, C. Y.; Letsiou, I.; Ozturk, I.; Eroglu, V.; Wilderer, P. A., Identification of Archaeal population in the granular sludge of an UASB reactor treating sewage at low temperatures. *J. Environ. Sci. Health A* **2008,** *43*, 1504–1510.
3. Gu, L.; Li, Q.; Quan, X.; Cen, Y.; Jiang, X., Comparison of nanosilver removal by flocculent and granular sludge and short- and long-term inhibition impacts. *Water Res.* **2014,** *58*, 62–70.
